# Mechnetor: a web server for exploring protein mechanism and the functional context of genetic variants

**DOI:** 10.1093/nar/gkab399

**Published:** 2021-06-02

**Authors:** Juan Carlos González-Sánchez, Mustafa F R Ibrahim, Ivo C Leist, Kyle R Weise, Robert B Russell

**Affiliations:** BioQuant, Heidelberg University, Heidelberg 69120, Germany; Biochemistry Center (BZH), Heidelberg University, Heidelberg 69120, Germany; BioQuant, Heidelberg University, Heidelberg 69120, Germany; Biochemistry Center (BZH), Heidelberg University, Heidelberg 69120, Germany; BioQuant, Heidelberg University, Heidelberg 69120, Germany; Biochemistry Center (BZH), Heidelberg University, Heidelberg 69120, Germany; BioQuant, Heidelberg University, Heidelberg 69120, Germany; Biochemistry Center (BZH), Heidelberg University, Heidelberg 69120, Germany; BioQuant, Heidelberg University, Heidelberg 69120, Germany; Biochemistry Center (BZH), Heidelberg University, Heidelberg 69120, Germany

## Abstract

Advances in DNA sequencing and proteomics mean that researchers must now regularly interrogate thousands of positional gene/protein changes in order to find those relevant for potential clinical application or biological insights. The abundance of already known information on protein interactions, mechanism, and tertiary structure provides the possible means to understand these changes rapidly, though a careful and systematic integration of these diverse datasets is first needed. For this purpose, we developed Mechnetor, a tool that allows users to quickly explore and visualize integrated mechanistic data for proteins or interactions of interest. Central to the system is a careful cataloguing of diverse sources of protein interaction mechanism, and an efficient means to visualize interactions between relevant and/or known protein regions. The result is a finer resolution interaction network that provides more immediate clues as to points of intervention or mechanistic understanding. Users can import protein, interactions, genetic variants or post-translational modifications and see these data in the best known mechanistic context. We demonstrate the tool with topical examples in human genetic diseases and cancer genomics. The tool is freely available at: mechnetor.russelllab.org.

## Graphical Abstract

**Graphical Abstract:**
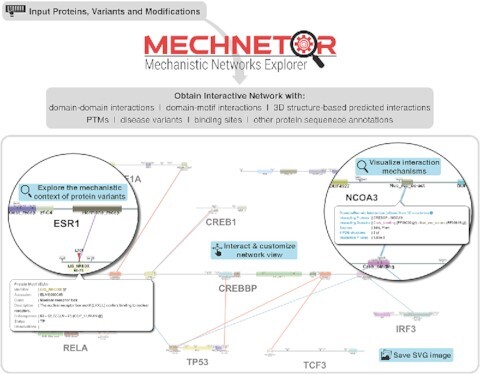
Overview of Mechnetor showing examples of interaction mechanisms overlayed with disease variants.

## INTRODUCTION

High-throughput sequencing technologies permit the identification of thousands of genetic variants in healthy and diseased individuals (1–3). However, understanding which among them are responsible for a disease, and more specifically, the molecular mechanisms by which such changes elicit disease pathology, remains challenging (4–6). Most popular methods for assessing variant impact (7,8) do not fully exploit available protein mechanistic data which makes them of limited use, for instance, when making clinical recommendations (9). Fortunately, there has been extensive recent growth in many functionally relevant datasets, including protein families (10), interactions (11,12), pathways (13), structures (14) and post-translational modifications (15,16). There are moreover constant improvements in protein functional annotations and information about previously studied variants (17). There is thus great potential to perform systematic mechanistic analyses of new genetic variants.

Because this wealth of information is scattered across numerous databases and the literature, gathering and integrating the data can be difficult and time-consuming. Effective data visualization is equally important as it allows a more rapid synthesis of diverse information into a coherent mechanism (18,19). Resources like Pfam (10), SMART (20) and InterPro (21) provide crucial details about protein functional modules and visual representations of protein modular architecture. However, these lack details regarding interactions, and one cannot readily visualize multiple proteins. On the other hand, resources for interrogating protein-protein interactions (PPIs), such as BioGRID (12), STRING (22) or GeneMANIA (23), do not specify protein segments involved in specific interactions. This information is stored in resources like 3did (24), a database of domain–domain (DDIs) and domain–motif interactions (DMIs) derived from 3D structures; or ELM (25), a database of linear motifs and their interaction domains with a related tool, iELM (26), for viewing them inside PPI networks.

There are many methods to assess the impact of variants on protein function, for example, based on sequence, phylogenetic and/or structural information (Polyphen-2 (8), MutationAssessor (27)); or using 3D structures to evaluate protein stability, folding or dynamics (FoldX (28), Rossetta (29)). These tools are useful for estimating the impact of collections of mutations generally on proteins, but do not normally consider the wider, protein-network context. Other tools, such as Mechismo (30) or dSysMap (31), help understand mechanism of action by combining structure with interaction data to predict and visualize the effect on interaction interfaces of known structure, though are limited in their reporting of mechanistic details lacking coordinate information.

Here, we present Mechnetor (mechnetor.russelllab.org), a new web tool that sits between the above resources. For sets of proteins, interactions and/or variants, Mechnetor quickly integrates diverse mechanistic data sources (PPIs, DDIs, DMIs, 3D structure, post-translational modifications, and numerous functional annotations) and constructs an interactive network for intuitive visualization of protein mechanisms. Proteins are represented as linear arrangements of domains, motifs and other functional elements, which permits the display of interactions between the relevant/known protein regions. The result is a finer resolution interaction network that enhances mechanistic interpretations of biological processes and variants of interest.

## RESULTS AND DISCUSSION

### Mechnetor: mechanistic networks explorer

**Figure 1.**
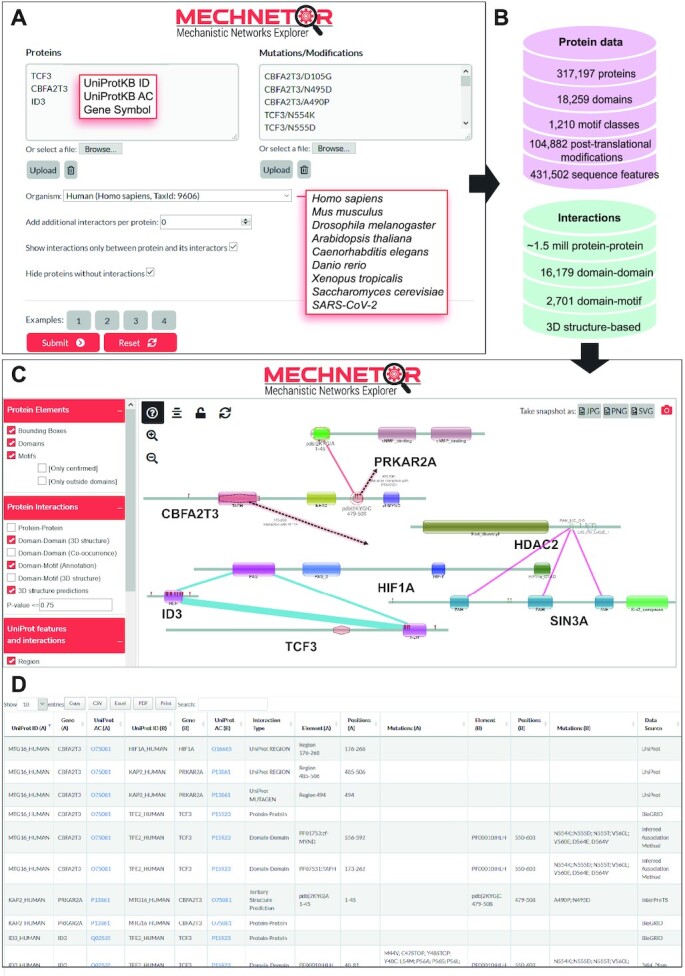
Overview of the Mechnetor web server. (**A**) Initial query submission page, where users can input proteins, protein pairs and/or protein variants for eight model organisms, plus SARS-CoV-2. (**B**) Summary of the different data sources present in Mechnetor's database, which include protein sequence features (domains, linear motifs, PTMs, and other functional regions) and interaction/associations between proteins (protein-protein) and their features (domain-domain, domain-motif, etc). (**C**) First component of the results page: the mechanistic network, containing query proteins and variants together with all gathered mechanistic information. Positional protein features are displayed along their sequences, while edges represent different types of interactions between them. Users can manually explore the network making use of many interactivity options. (**D**) The second component of the results page is a table containing all interactions between any two proteins comprised in the network.

Mechnetor is a web tool that allows for a quick and user-friendly exploration of proteins and variants of interest within a detailed mechanistic context. The general workflow is represented in Figure 1. Users can directly input proteins or protein pairs in the form of UniProtKB accessions and identifiers, or gene symbols (with the option of automatically adding any number of known interactors for those proteins); as well as their own sets of genetic variants and/or post-translational modifications (Figure 1A). Currently, Mechnetor supports eight of the most common model organisms: *Homo sapiens*, *Mus musculus*, *Danio rerio*, *Xenopus tropicalis, Arabidopsis thaliana*, *Drosophila melanogaster*, *Caenorhabditis elegans*, and *Saccharomyces cerevisiae*. In addition, we included SARS-CoV-2 proteins, which can be queried together with human proteins. We will add a more complete set of human viral proteins in the future.

For each query protein, Mechnetor systematically gathers domains, linear motifs, post-translational modifications (from various data sources; see Materials and Methods) and other relevant sequence features (from UniProt), as well as interactions between those proteins (PPIs) and their elements (DDIs and DMIs), interactions predicted from 3D structures, and interactions or associations extracted from annotations. This process relies on an underlying database where data from diverse sources are carefully integrated, by matching different formats and descriptors, ensuring that information can be retrieved efficiently (Figure 1B).

The collected data are then used to create an interactive mechanistic network that can include variants/PTMs mapped into their corresponding protein positions, and is presented to the user together with extensive interactivity options to facilitate an intuitive exploration (Figure 1C). Additionally, a searchable table lists in detail all interaction evidence contained in the network, and can be also downloaded in text format for local analysis (Figure 1D). The results page can be bookmarked for later access–results will be kept for a period of no less than a month.

### The mechanistic network

**Figure 2.**
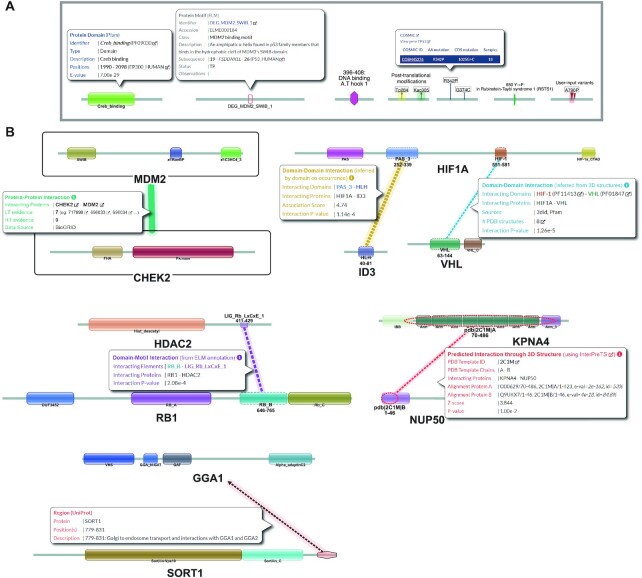
Illustrative examples of network components. (**A**) Examples of functional elements extracted from different proteins for illustration. For some, a popup box with additional information (that can be toggled on when clicking on the node) is displayed. From left to right: protein domain (from Pfam); linear motif (from ELM); DNA binding site (from UniProt); phosphorylation and acetylation sites (from UniProt and PhosphositePlus); cancer missense variants (from COSMIC); genetic disease variant (from UniProt); variants input by the user. (**B**) Selected pairs of human proteins to illustrate the different types of interactions (with popup boxes). From top to bottom, and left to right: protein–protein interaction (green) between *MDM2* (UniProtKB: Q00987) and *CHEK2* (UniProtKB: O96017) linking the entire proteins, supported by seven low-throughput experiments (according to BioGRID); domain-domain interaction inferred by domain co-occurrence (yellow) between the *Pas_3* domain of *HIF1A* (UniProtKB: Q16665) and the *HLH* domain of ID3 (UniProtKB: Q02535), with an association score of 4.74; domain-domain interaction (cyan) between the *HIF-1* domain of *HIF1A* and the *VHL* domain of *VHL* (UniProtKB: P40337) supported by eight PDB structures (according to 3did and also Pfam); domain-motif interaction (purple) between the *RB_B* domain of *RB1* (UniProtKB: P06400) and a *LIG_Rb_LxCxE_1* (ELM accession: ELME000007) in *HDAC2* (UniProtKB: Q92769) (from ELM); interaction predicted through tertiary structure (red) between *KPNA4* (UniProtKB: O00629) and *NUP50* (UniProtKB: Q9UKX7) using InterPreTS and PDB ID: 2C1M as template; interaction (black and orange) between a region of *SORT1* (UniProtKB: Q99523) and *GGA1* (UniProtKB: Q9UJY5) extracted from UniProt annotations.

The Mechnetor network view shows detailed mechanistic information for every submitted protein. Proteins are represented as linear diagrams (length proportional) of functional elements (Table 1; Figure 2A). Edges in the network specifically link entire proteins or the functional elements involved in different types of interactions, which are coloured accordingly (Table 2; Figure 2B). Some of them are weighted according to particular parameters (e.g. number of experimental studies, number of 3D structures, etc) to indicate the extent of interaction evidence, which is reflected in edge thickness. Interactions involving domains and motifs are given a *P*-value that indicates the probability of randomly observing the particular pair in the interactome of the organism considered.

**Table 1.** Mechnetor network protein components (nodes)

Protein component	Description	Source
Domains	Domain architecture	Pfam
Motifs	Linear motifs involved in potential interactions with domains present in the network. Toggles allow to only show confirmed motif instances and exclude motifs inside protein domains	ELM, 3did
UniProt sequence features	These include diverse annotations, such as regions of interest, binding sites for chemical groups, metals and DNA, transmembrane regions, disulphide bonds, and sites altered by mutagenesis experiments	UniProt
Post-translational modifications	Phosphorylation, acetylation and glycosylation sites	UniProt, PhosphositePlus
Variants/Modifications	Variants and modifications input by the user	User input
Disease variants	Variants involved in human genetic diseases	UniProt
Cancer variants	Cancer missense variants in human	COSMIC

**Table 2.** Mechnetor network interactions types (edges)

Interactions	Description	Source
Protein-protein	Represents current experimental evidence for the interaction between two proteins	BioGRID
Domain-domain (i)	Domain interactions inferred from 3D structures directly or through homology	3did
Domain-domain (ii)	Domain interactions inferred by significant co-occurrence of domain pairs in known interacting proteins. A log-odds indicates the strength of the domain association.	Predicted (see Methods)
Domain-motif (i)	Interactions between linear motifs and their binding domains, obtained from annotated motif classes. Certain restrictions are applied based on annotation to ensure these interactions are biologically significant (see Methods)	ELM
Domain-motif (ii)	Interactions between linear motifs and their binding domains, inferred from 3D structure	3did
3D-based	Links potential interfaces predicted through tertiary structure. Uses own scoring system	InterPreTS
Other associations	Associations between certain UniProt features (regions, binding sites, mutagenesis) and other proteins in the network	UniProt

Users can click on any protein element or interaction to display a box with more information and links to original data sources (Figure 2). The control panel allows for all nodes and edges to be toggled on or off individually or by setting thresholds (for interactions). Initial protein positions in the network are completely arbitrary, and they can be moved freely by simply dragging them. The graph viewer also allows to zoom in or out without any loss of image quality. All these interactivity options permit users to explore the data in the network, but also to customize the view which can be exported at any time as a snapshot image (PNG or JPEG), or vector graphics (SVG) containing the full network, suitable for editing and preparing publication-quality figures.

The interactive network is especially designed to investigate mechanistic details for only a handful of proteins at the time. The ‘hairball’ effect is an intrinsic problem of network visualization and we do not recommend (or indeed allow) networks involving >20 proteins. The user will be warned if the input is too big, and the network will contain only a subset of proteins. However, the table will still contain all relevant data gathered for the complete input protein set.

### Case studies

**Figure 3.**
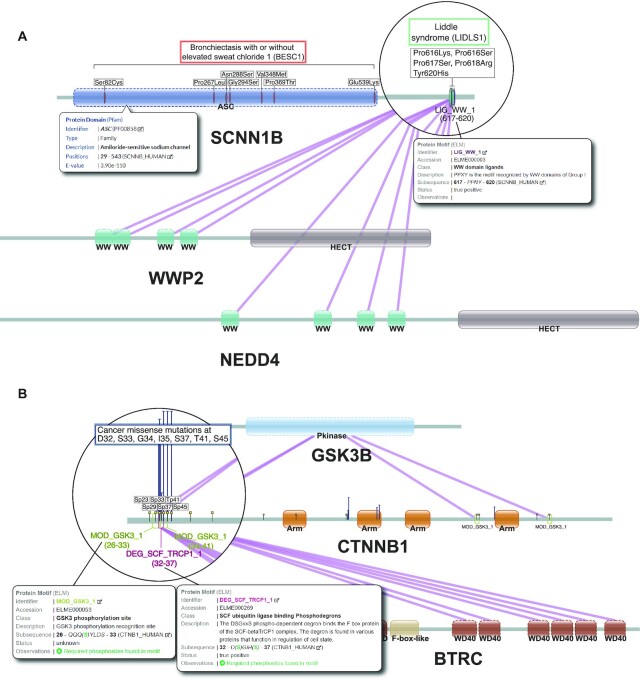
Case studies illustrating Mechnetor's functionalities. (**A**) Network view of *SCNN1B* (UniprotKB: P51168), *WWP2* (UniProtKB: O00308) and *NEDD4* (UniProKB: P46934), showing domain architecture, domain-motif interactions from ELM (purple lines) and UniProt's genetic disease variants (bronchiectasis variants in orange and Liddle syndrome variants in green). *SCNN1B* C-terminal region has been zoomed to enhance visualization of the overlap of Liddle syndrome variants and the *LIG_WW_1* motif. Popup boxes show annotation for the *ASC* domain (left), and the *LIG_WW_1* motif in *SCNN1B* (right) which contains its sequence on the protein, supporting the observation that Liddle syndrome variants (amino acid substitutions in Pro-617, Pro-618 and Tyr-620) alter the motif. (**B**) Network view of *CTNNB1* (UniProtKB: P35222), *GSK3B* (UniProtKB: P49841) and *BTRC* (UniProtKB: Q9Y297), displaying protein domains, domain-motif interactions from ELM (purple lines), phosphorylation sites (small yellow flags), and cancer missense variants from COSMIC (blue t-shaped lines, heights are proportional to number of samples). We set a minimum number of 5 samples for cancer variants to be displayed. The N-terminal region of *CTNNB1* (zoomed) shows the overlap between a cluster of cancer variants, a number of phosphosites and three motifs: two *GSK3B* recognition sites (*MOD_GSK3*) and the diphospho-degron (*DEG_SCF_TRCP_1*) recognized by *BTRC*. Popup boxes show more detailed annotations and let us know that the required phosphosites are found within these motifs. This support the validity of these motifs and suggests that cancer variants in this positions might result in the disruption of both recognition mechanisms.

We interrogated a dataset of Mendelian disease variants (17) and found instances where mechanistic differences highlighted by Mechnetor correspond to different pathologies. For instance, protein *SCNN1B* (UniprotKB: P51168) constitutes the β subunit of the heterotrimeric epithelial sodium channel *ENaC*, located mostly in high resistance epithelia cells in vertebrates, and involved in maintaining homeostasis and regulating blood pressure (32). Variants in this protein are related to two genetic diseases: Bronchiectasis with or without elevated sweat chloride 1 (BESC1) and Liddle syndrome 1 (LIDLS1). A quick glance at the network display reveals that variants for these two diseases are located in different regions of the protein (Figure 3A). BESC1 variants are more widespread but all within the most conserved part of the protein which comprises the sodium channel (*ASC* family domain [Pfam accession: PF00858]). Thus, they are more likely deleterious and result in decreased channel activity (33). In contrast, LIDLS1 variants are clustered in a small region towards the C-terminus that overlaps with a WW domain binding motif (LIG_WW_1 or ELM accession: ELME000003). The nature of these amino acid changes suggest that LIDLS1 variants disrupt the motif pattern, thus affecting recognition of *SCNN1B* by E3 ubiquitin ligases, like *WWP2* or *NEDD4*. This would result in a decrease of ubiquitination, which in turn would impair degradation of the *EnAC*, that would remain constitutively active, resulting in an increase of blood volume and pressure, and this is in fact the known molecular mechanism causing LIDLS1 (34,35).

To demonstrate how the tool can study somatic cancer variants, we considered the oncogene *CTNNB1* (UniProtKB: P35222). Using the preloaded cancer missense variant dataset from COSMIC (3), and requiring ≥5 samples for a variant to be reported, a clear hotspot of highly recurrent variants can be located at the N terminus of the protein, in a region that also contains several *GSK3B* (UniProtKB: P49841) phosphorylation sites (recognised by *MOD_GSK3_1* motifs [ELM accession: ELME000053]) targeted by these variants (S33, S37, T41 and S45; Figure 3B). There are other *MOD_GSK3* motifs that could be false positives owing to the simplicity of its pattern, which is essentially just a pair of Serine/Threonine residues separated by three amino acids (…[ST]…[ST]). Only a few, including the two within the cancer hotspot, actually correspond with known phosphosites supported with experimental evidence (obtained from UniProt and PhosphositePlus). Moreover, these phosphorylations are required for the recognition of *CTNNB1* by *BTRC* (UniProtKB: Q9Y297), a component of E3 ubiquitin-protein ligase complex, through a diphospho-dependent degron *DEG_SCF_TRCP1_1* (ELM accession: ELME000269) that interacts with its WD40 β-propeller. Therefore, these variants ultimately prevent ubiquitination of *CTNNB1* and its subsequent degradation, which can then translocate to the nucleus and continuously promote transcription of its target genes (36).

## MATERIALS AND METHODS

### Web server implementation

The Mechnetor web server is built with Python3 under the Flask micro-web framework, and uses a PostgreSQL database. Data visualization makes extensive use of the Cytoscape JavaScript library (cytoscape.js) (37).

### Data sources and processing

All data required by Mechnetor were obtained from publicly available data sources and stored in our PostgreSQL database after some pre-processing and integration, that ensures information can be quickly retrieved and displayed by the tool.

Protein names, identifiers, gene, sequences, and multiple other annotations (post-translational modifications, variants, mutagenesis experiments, functional and interacting regions, transmembrane regions, disulphide bonds and binding sites) are obtained from UniProt (17). All other data are always referenced to UniProt proteins. Protein domains are gathered from Pfam (10), or identified with the PfamScan tool (38) against the Pfam-A database with a 0.001 expectation value cut-off. Short linear motifs instances are obtained from ELM (25) and 3did (24), comprising 291 and 812 motif classes respectively, and their sequence patterns are used to identify potential new instances by regex matching. Additional PTMs are extracted from PhosphositePlus (15). Human cancer protein missense variants are obtained from COSMIC genome-wide screens only (3). Protein–protein interactions are gathered from BioGRID (12). 3did (24) is used as source of domains interactions. 3did systematically charts atomic contacts between Pfam domains within 3D structures. In addition, we predict domain-domain associations from interaction data (see below). Domain-linear motif interactions are obtained from 3did, and also derived from a modified dataset from ELM (see below). To predict 3D structure-based interactions and interfaces, Mechnetor runs an internal version of InterPreTS (39,40), which itself uses the RCSB PDB database (14). InterPReTs compares sequence pairs to proteins interactions of known structure and scores (*Z* score, *P*-value) how well the sequences fit on any identified interface.

Data will be periodically updated. Current data versions can always be consulted at: mechnetor.russelllab.org/help.

### Reviewing domain–motif interactions

The source file of ELM interaction domains data (elm.eu.org/interactiondomains) only lists motif classes and their interaction domains but, actually, not all motifs that interact with the same domain type can interact with the same proteins. For example, ELM has >30 motif classes that interact with the ‘protein kinase domain’ (Pfam identifier: PF00069), which in the human proteome is present in hundreds of proteins. However, most of these motif classes are only recognized by the kinase domains of very particular and different protein kinases, e.g. the ELM motif *MOD_NEK2_1* is the specific phosphorylation site of the Serine/Threonine-protein kinase *NEK2*, while *DOC_MAPK_gen_1* is the docking motif of members of the MAP kinase family (MAPKs). Furthermore, some motifs are exclusively located in certain proteins, such as *LIG_PEX14_1* which mediates the interaction between *PEX5* and *PEX14;* or are exclusive to certain taxa, like *LIG_PAM2_2*, which is a variant of the PABP (Poly-adenylate binding protein)-interacting motif specific for animals.

All this information and more can be found in the curated entries of motif classes at the ELM website. Based on this, we manually annotated each motif-domain interaction with additional restrictions and requirements for the interaction to take place. These include: restriction to certain taxa, restriction of interaction domain and/or motif to only certain genes, require the presence of other linear motifs in the same protein, and require the presence of phosphosites within the motif. An updated version of these ELM interaction domains/proteins with our additional annotations can be found in Supplementary File S1. In order to present only the most biologically relevant information, Mechnetor only shows domain-motif interactions that match to protein/domain motif pairs in this revised table.

### Scoring and inferring DDI interactions

To infer interactions between protein domains, we use the method first described by Sprinzak and Margalit in 2001 (41) for the identification of over-represented sequence-signatures pairs in interacting proteins by comparing their expected and observed frequencies. This is done for each organism independently, using a subset of non-redundant PPI reported by at least two experimental sources. For every possible domain-domain combination, the method compares the observed number of interacting proteins containing the pair of domains (one or more times) against the expected number according to the individual frequencies of the domains, and assess the significance returning a log-odds or *association score*. A high log-odds value indicates a strong correlation between the corresponding domains in interacting proteins. We define a domain pair as enriched if its association score is greater than or equal to 2, but to avoid not significant associations, only if its observed count is also greater than or equal to 4, and the individual counts of proteins containing each of the signatures are greater than or equal to 4. We assigned an association score of –5 (which is smaller than the minimum log-odds value calculated) to those pairs where the observed frequency was zero. In addition, the observed frequency of every domain–domain, but also domain-motif pair, is also reported as a *P*-value for every DDI and DMI. It represents the probability of finding the particular pair in the interaction dataset and thus can be used to estimate their significance.

## CONCLUSION

Despite the immense volume of data generated by sequencing efforts, its impact on the advancement of medical knowledge and the development of patient-tailored treatments has been limited by our still narrow ability to interpret the molecular consequences of coding variants. This task necessarily requires the simultaneous analysis of diverse protein data, which often implies consulting several data sources and applying computational approaches to take further advantage of them.

Mechnetor facilitates this by performing a systematic and fast integration of diverse protein data and presenting it to the user in an interactive and intuitive way. One of our priorities was to make this tool very user-friendly so, in essence, Mechnetor can be used by simply entering a pair of proteins and/or protein variants, clicking the submit button, and getting mechanistic ideas in a few seconds. Further possibilities include studying larger datasets of interacting protein pairs by directly downloading the integrated data for local analysis. We will update Mechnetor data regularly, and we plan on supporting more organisms, as well as extending some of its functionalities.

## DATA AVAILABILITY

Mechnetor is a web server freely accessible without login requirement at mechnetor.russelllab.org. The source code is available at https://github.com/JCGonzS/mechnetor.

## Supplementary Material

gkab399_Supplemental_Files

## Notes

Present address: Ivo C. Leist, CNAG-CRG, Centre for Genomic Regulation, Barcelona Institute of Science and Technology, Barcelona 08028, Spain.
